# Qualification rate and associated factors regarding COVID-19 clinical skills training based on scenario simulation teaching to medical staffs in China: a hospital-based cross-sectional study

**DOI:** 10.1186/s12909-024-05733-3

**Published:** 2024-07-19

**Authors:** Manyuan Cai, Yanping Chen, Fangting Luo, Yanqun Zheng, Ying Liu, Bing Xiao, Xiaoyan Wang, Lulu Sun, Yi Lin, Xianhu Zeng, Shuni Tan, Ke Liu, Yuanbo Gu, Jinghua Wang, Xianjia Ning, Jing Yuan, Min Wen, Jing Cao

**Affiliations:** 1 Department of Scientific Research and Teaching (Clinical Skills Simulation Training Center), Shenzhen Third People’s Hospital and The Second Affiliated Hospital, School of Medicine, Southern University of Science and Technology, Shenzhen, Guangdong 518112 China; 2 Department of Difficult and Severe Liver Disease, Shenzhen Third People’s Hospital and The Second Affiliated Hospital, School of Medicine, Southern University of Science and Technology, Shenzhen, Guangdong 518112 China; 3 Clinic, Shenzhen Third People’s Hospital and The Second Affiliated Hospital, School of Medicine, Southern University of Science and Technology, Shenzhen, Guangdong 518112 China; 4 Department of Preventive Health Care and Hospital Infection Control, Shenzhen Third People’s Hospital and The Second Affiliated Hospital, School of Medicine, Southern University of Science and Technology, Shenzhen, Guangdong 518112 China; 5 Gastrointestinal Endoscopy Center, Shenzhen Third People’s Hospital and The Second Affiliated Hospital, School of Medicine, Southern University of Science and Technology, Shenzhen, Guangdong 518112 China; 6 Department of Spine Surgery, Shenzhen Third People’s Hospital and The Second Affiliated Hospital, School of Medicine, Southern University of Science and Technology, Shenzhen, Guangdong 518112 China; 7 Department of Pediatrics, Shenzhen Third People’s Hospital and The Second Affiliated Hospital, School of Medicine, Southern University of Science and Technology, Shenzhen, Guangdong 518112 China; 8 Two Department of Pulmonary Diseases, Shenzhen Third People’s Hospital and The Second Affiliated Hospital, School of Medicine, Southern University of Science and Technology, Shenzhen, Guangdong 518112 China; 9 Department of Hepatitis and Cirrhosis, Shenzhen Third People’s Hospital and The Second Affiliated Hospital, School of Medicine, Southern University of Science and Technology, Shenzhen, Guangdong 518112 China; 10 Fever Clinic, Shenzhen Third People’s Hospital and The Second Affiliated Hospital, School of Medicine, Southern University of Science and Technology, Shenzhen, Guangdong 518112 China; 11 Center of Clinical Epidemiology, Shenzhen Third People’s Hospital and The Second Affiliated Hospital, School of Medicine, Southern University of Science and Technology, Shenzhen, Guangdong 518112 China; 12 Department of Infectious disease, Shenzhen Third People’s Hospital and The Second Affiliated Hospital, School of Medicine, Southern University of Science and Technology, Shenzhen, Guangdong 518112 China; 13 Department of Nursing, Shenzhen Third People’s Hospital and The Second Affiliated Hospital, School of Medicine, Southern University of Science and Technology, 29 Bulan Road, Longgang District, Shenzhen, Guangdong Province 518112 China

**Keywords:** Clinical skills training, Infectious diseases, Scenario Simulation, Education, Gender, Professional background

## Abstract

**Background:**

The coronavirus disease (COVID-19) pandemic has accentuated the need for effective clinical skills training in infectious diseases. This study aimed to explore the influencing factors of infectious disease clinical skills training based on scenario simulation teaching for medical staff in China.

**Methods:**

This hospital-based, cross-sectional study was conducted at the Third People’s Hospital of Shenzhen between March and December 2022. Scenario simulation teaching was applied, and factors such as gender, educational level, professional background, and previous experience were examined to determine their impact on qualification outcomes.

**Results:**

The study included participants primarily between the ages of 20–40 years, with a higher proportion of women holding university degrees. Nurses and physicians were more likely to qualify, indicating the significance of professional backgrounds. Women showed a higher likelihood of qualifying than men and higher educational attainment correlated with better qualification rates. Prior experience with protective clothing in isolation wards was a significant determinant of successful qualification. Multivariate analysis underscored the influence of sex, education, and previous experience on training effectiveness.

**Conclusion:**

Scenario simulation is an effective strategy for training clinical skills in treating infectious diseases. This study highlights the importance of considering sex, education, professional background, and prior experience when designing training programs to enhance the efficacy and relevance of infectious disease training.

## Introduction

Since the COVID-19 outbreak in 2020, COVID-19 has profoundly affected the global health, healthcare systems, societies, and economies. As of September 24, 2023, the pandemic had infected over 770 million people worldwide, with a death toll exceeding 69 million [1]. The World Health Organization reported that by the end of 2021, there would have been approximately 14.9 million excess deaths attributable to COVID-19 [2]. These staggering numbers highlight the critical need for effective healthcare strategies, particularly clinical skill training for infectious diseases.

Clinical skills training for infectious diseases is of paramount importance in the medical field. Current research underscores the importance of equipping healthcare professionals with the skills necessary to effectively diagnose, treat, and manage infectious diseases [3]. Studies show that peer learning in skills laboratories offers significant advantages, potentially enhancing the quality of undergraduate medical education, especially given the limited availability of teaching resources [4]. The integration of didactic dermatological teaching with practical clinical skills sessions has been shown to solidify learning [5].

Simulation training has emerged as a key tool in teaching both clinical and non-clinical skills. The direct impact on improving patient care in mental health teaching has been particularly notable [6]. Comparative studies between situational simulation education and traditional clinical education underscore the potential of the former in enhancing clinical skills [7–9]. In particular, scenario-based simulation teaching, an innovative approach to medical education, has gained significant attention. This method offers medical students and healthcare professionals a more realistic training experience by simulating real-world patient scenarios. This approach allows learners to practice and enhance clinical skills in a relatively safe environment while also gaining a deeper understanding of complex cases [10, 11]. However, few reports address the application of situational simulation teaching in clinical skills training of infectious diseases. For instance, using simulation-based training program for Ebola personal protective equipment training could be an effective and practical method of developing competent all healthcare workers in Ebola personal protective equipment [12]. In addition, results from a Hong Kong study suggest that a hospital-wide, multidisciplinary simulation-based training in infection control minimizes in-hospital transmission of COVID-19 in this highly stressful and time-sensitive high-risk procedure [13].

The effect of scenario-based simulation teaching has also been demonstrated in many medical disciplines in China [14–17]. However, application in the field of infectious diseases remains limited. A few studies from China have confirmed that occupational protection knowledge simulation-based training can significantly improve the theoretical level and skill level of medical personnel [18–20].

A previous study suggested that an objective structured clinical examination (OSCE) model-based scenario simulation teaching method is as an effective approach to train young nurses on fever prevention and control during the COVID-19 outbreak. It illustrates the practical adaptations and the immediate applicability of scenario-based simulations in acute outbreak settings [21]. A recent report incorporated self-efficacy and diversified training into the assessment system and underscored the importance of personalized and adaptive training strategies within scenario-based simulation frameworks [22]. However, factors influencing the effect of the scenario-based simulation teaching on clinical skills training in infectious diseases have not been fully explored in China, especially trainee-related factors, such as demographic characteristics, educational background, and professional experience. Because these factors influence the effectiveness of simulation-based training, studying them is essential for developing training programs to meet the diverse needs of health workers and to improve the quality of health care during infectious disease outbreaks.

Thus, this study aims to examine the application of scenario-based simulation teaching in clinical skill training for medical staff in infectious disease departments, as well as to identify factors that may impact training outcomes.

## Materials and methods

### Study design and subjects

This hospital-based cross-sectional study was conducted at the Third People’s Hospital of Shenzhen, a specialized infectious disease hospital, between March and December 2022. The participants were medical staff, who were defined as qualified health technicians at all levels and of various types who possessed the corresponding qualifications and professional certificates following assessment and approval and recognition by the health administrative department, including physicians, nurses, pharmacists, and other medical technicians. Excluded from the study were resident physicians, visiting scholars, interns from our hospital, and individuals unwilling to participate; individuals with factors such as leave of absence, sickness, and pregnancy were also excluded.

### Training program

The training program, tailored for frontline personnel combatting the COVID-19 epidemic, was guided by the latest national COVID-19 Prevention and Control Guidelines, which aimed to impart essential knowledge and skills, including the use of personal protective equipment, hospital infection protection protocols, and management practices for isolation wards.

This program utilized a blended learning approach, combining online theoretical lessons with practical on-site training sessions. The essential aspects of the training included online video lessons provided through the hospital’s digital platform, focusing on theoretical knowledge and demonstration of technical operations, and scheduled on-site practical sessions in a dedicated training area, designed to mimic real-world scenarios where trainees applied their learned skills under direct supervision.

**Fig. 1:**
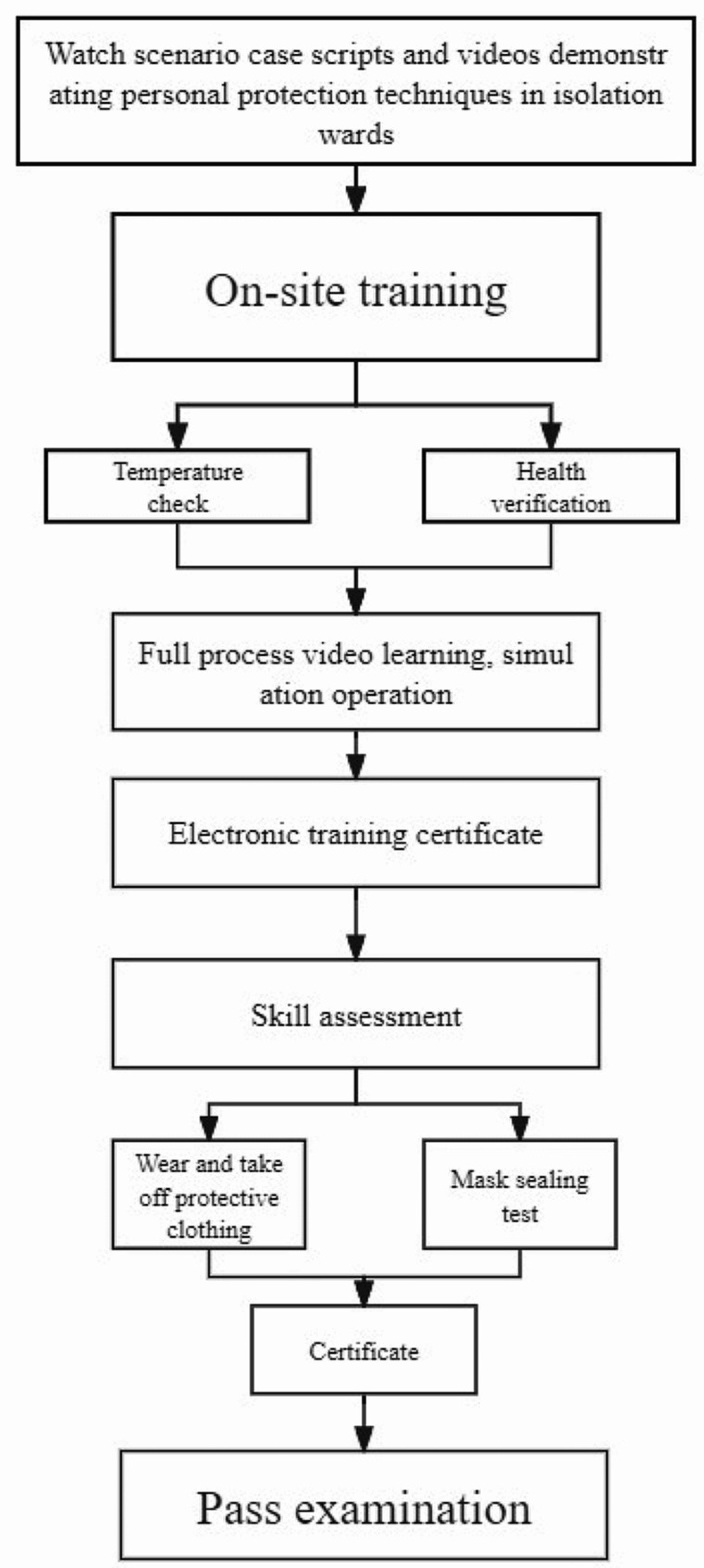
Training program First, the scenario case scripts and isolation ward personal protection technical operation demonstration videos were created and made accessible through the hospital’s CCMTV Cloud Housekeeper online platform. After completing the video lessons, the trainees participated in scheduled on-site training and assessments, including temperature checks and health status verification. Onsite training took place in a specially designed area featuring three zones and two channels, with detailed guidance and hands-on practice overseen by the instructors. Upon completing the onsite training, the trainees underwent assessments of protective equipment procedures, operational standards, and isolation ward layouts, with a maximum score of 100 points. Those who failed the assessment were provided with additional practice and guidance

First, scenario case scripts and technical operation demonstration videos on personal protection in the isolation ward were created and made accessible through the hospital’s CCMTV Cloud Housekeeper online platform. After completing the video lessons, the trainees participated in scheduled on-site training and assessments, including temperature checks and health status verification. Onsite training took place in a specially designed area featuring three zones and two channels, with detailed guidance and hands-on practice overseen by instructors. Upon completing the onsite training, the trainees underwent assessments of protective equipment procedures, operational standards, and isolation ward layouts, with a maximum score of 100 points. Those who failed the assessment were provided with additional practice and guidance (Fig. 1).

A specialized training and assessment management group was formed with members from various departments, including infection management, nursing, medical affairs, and the Clinical Skill Simulation Training Center. This group, which was divided into five subgroups (training, assessment, mask airtightness test, certification, data collation, and material management), was responsible for flexible staffing and regular member training based on the updated disease prevention guidelines.

The training program was divided into seven modules, each designed to address different aspects of infectious disease management with a specific emphasis on COVID-19. The modules combined theoretical knowledge with practical exercises, ensuring comprehensive skill development among the participants. Table 1 provides a clear and detailed overview of the training content and outlines the specific topics covered during the training, including but not limited to the correct use of personal protective equipment, strategies for minimizing occupational exposure, and protocols for the management of isolation wards. The training was divided into modules, each focusing on different aspects of infectious disease control with a special emphasis on COVID-19. The duration of each module, along with the total training time, is also specified in this table.

**Table 1:** Outline of program for frontline personnel protective skill training during the COVID-19 epidemic

Module	Focus Area	Duration
**1**	Introduction to COVID-19	1 h
**2**	Use of Protective Equipment	2 h
**3**	Hand Hygiene and Disinfection Practices	45 min
**4**	Operational Standards in Isolation Wards	1 h
**5**	Management of Exposure Incidents	30 min
**6**	Psychological Support for Healthcare Workers	45 min
**7**	Simulation Exercises: Applying Knowledge in Practice	2 h
**Total**		8 h

**Assessment of Training Effectiveness**The effectiveness of the training was evaluated based on the trainees’ performance in applying protective equipment procedures, adherence to operational standards, and understanding of isolation ward layouts. Participants underwent a structured assessment post-training, with additional support offered to those requiring further practice.

### Evaluation of tool’s criterion

The evaluation tool was formulated according to the two standards promulgated by the state: the Expert consensus on personal protection at work in different regions of medical institutions during the COVID-19 epidemic [23] and Technical Guidelines for the Prevention and Control of Novel Coronavirus Infection in Healthcare Settings [24].

The evaluation criteria of this study are The analysis of the reliability and validity of the evaluation tools were not performed in the special period of the outbreak of the new champions league, because the time is pressing.

### Statistical analysis

Continuous variables are presented as means ± standard deviation (SDs) and were compared using Student’s t-tests. Categorical variables are expressed as numbers and frequencies and were compared using chi-squared tests. Logistic regression analyses were used to explore the independent predictors of unqualified training, with the results expressed as odds ratios (ORs) and 95% confidence intervals (CIs). In the multivariate analysis, the independent variables were selected from those covariates with statistical differences in the univariate analysis. All analyses were performed using SPSS (version 25.0, Chicago, IL, USA), with *P* < 0.05 indicates statistical significance.

## Results

**Fig. 2:**
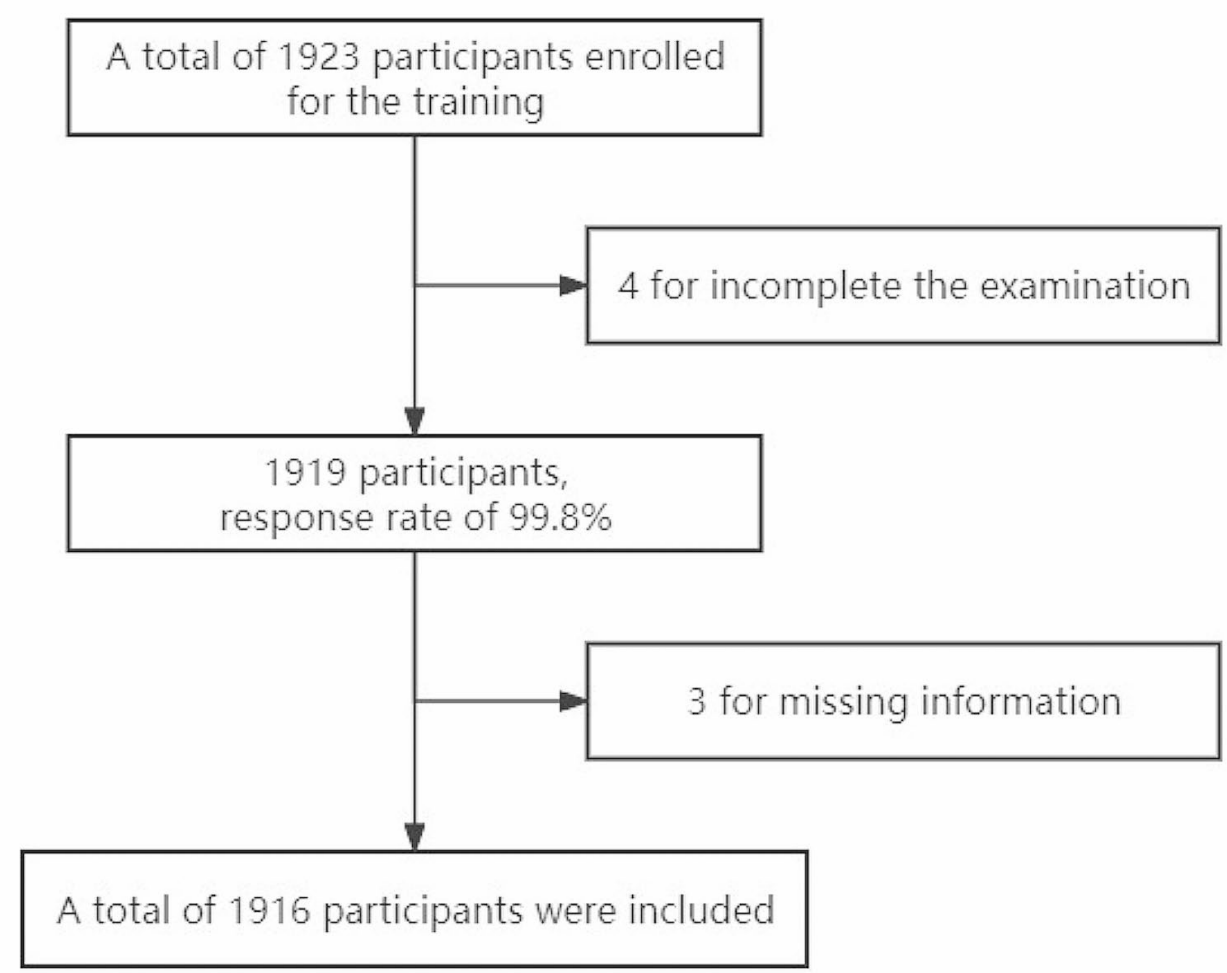
Flow chat of participants’ selection A total of 1923 participants were trained and took part in this study. After four participants were excluded for falling to complete the examination, the response rate was 99.8%. Moreover, three participants had missing information, leaving a final total of 1916 participants who were analyzed

A total of 1923 participants were trained and took part in this study. After four participants were excluded for falling to complete the examination, the response rate was 99.8%. Moreover, three participants had missing information, leaving a final total of 1916 participants who were analyzed (Fig. 2).

### Demographic and professional characteristics of participants

Of these 1916 participants, 65.7% were female (*n* = 1259). The demographic profiles of the participants predominantly fell into the age groups of 20–30 (42.5%) and 31–40 (37.6%) years. Gender disparity was apparent, with 48.8% of female participants in the 20–30-year age group compared to 30.4% of male participants, who were mainly represented in the 31–40-year age group (41.4%). Most participants had university degrees (56.0%), with a higher proportion of female participants (60.2%) holding university degrees than their male counterparts (47.9%). The distribution of professionals was diverse, of which physicians (32.0%) and nurses (49.8%) were the most prevalent. On average, male participants had a BMI of 23.91 kg/m², whereas female participants had a BMI of 21.29 kg/m², with the majority classified as normal weight (78.0%). A significant majority (79.4%) reported wearing protective clothing in the isolation wards. All participants voluntarily participated in the isolation ward work and had a history of training. The median working time was slightly different by gender, with men averaging 9.00 h, women 6.00 h, and an overall median of 7.00 h (Table 2).

**Table 2:** Characteristics of participants at baseline

Characteristics	Men	Women	Total
**cases, n (%)**	657 (34.3)	1259 (65.7)	1916 (100)
**Age group, n (%)**			
**20–30 yrs**	200 (30.4)	615 (48.8)	815 (42.5)
**31–40 yrs**	272 (41.4)	449 (35.7)	721 (37.6)
**41–50 yrs**	127 (19.3)	151 (12.0)	278 (14.5)
**51–60 yrs**	58 (8.8)	44 (3.5)	102 (5.3)
**Education level, n (%)**			
**College**	70 (10.8)	239 (19.2)	309 (16.3)
**University**	310 (47.9)	750 (60.2)	1060 (56.0)
**Master degree**	204 (31.5)	230 (18.5)	434 (22.9)
**Doctor degree**	63 (9.7)	26 (2.1)	89 (4.7)
**Career, n (%)**			
**Doctors**	359 (55.4)	248 (19.9)	607 (32.0)
**Technicians**	95 (14.7)	68 (5.4)	163 (8.6)
**Nurses**	123 (19.0)	820 (65.8)	943 (49.7)
**Others**	58 (9.0)	67 (5.4)	125 (6.6)
**Administrator**	13 (2.0)	45 (3.6)	58 (3.1)
**BMI, Kg/m**^**2**^, **means (SD)**	23.91 (3.02)	21.29 (2.84)	22.17 (3.15)
**BMI group, n (%)**:			
**Normal weight**	401 (61.0)	1094 (86.9)	1495 (78.0)
**Over weight**	200 (30.4)	136 (10.8)	336 (17.5)
**Obesity**	56 (8.5)	29 (2.3)	85 (4.4)
**Experience of wearing and taking off protective clothing in isolation ward, n (%)**:			
Yes	539 (77.5)	1013 (80.5)	1522 (79.4)
No	148 (22.5)	246 (19.5)	394 (20.6)
**Voluntarily working in the isolation ward, n (%)**:			
Yes	657 (100)	1259 (100)	1916 (100)
No	0	0	0
**History of participation training previous, n (%)**:			
Yes	657 (100)	1259 (100)	1916 (100)
No	0	0	0
**Working times, median (min, max)**:	9.00 (0, 37)	6.00 (0, 38)	7.00 (0, 38)

### Baseline characteristics by examination result

Table 3 delineates a significant difference in qualification status based on sex (*P* < 0.001), with women exhibiting a higher qualification rate (74.0%) than men (65.0%). Age did not significantly affect the qualification rate (*P* = 0.845), suggesting a consistent qualification rate across age groups. Educational level was a notable factor; individuals with bachelor’s and master’s degrees were more likely to qualify than those with college education (*P* = 0.042). A comparison of professional backgrounds revealed significant differences (*P* = 0.001), with nurses and physicians showing the highest qualification rates. The average BMI was slightly greater in the unqualified group (22.46) than in the qualified group (22.04; *P* = 0.013). BMI categories showed slight, non-significant differences in qualification rates (*P* = 0.095). Prior experience with protective clothing was significantly associated with qualification status (*P* < 0.001). There were no significant differences in qualification rates based on median working time (*P* = 0.210).

**Table 3:** Characteristics of participants at baseline

Characteristics	Qualified	Unqualified	*P*
**Sex:**			**< 0.001**
**Men**	427 (65.0)	230 (35.0)	
**Women**	932 (74.0)	327 (26.0)	
**Age group, n (%)**			0.845
**20–30 yrs**	580 (71.2)	235 (28.2)	
**31–40 yrs**	508 (70.5)	213 (29.5)	
**41–50 yrs**	195 (70.1)	83 (29.9)	
**51–60 yrs**	76 (74.5)	26 (25.5)	
**Education level, n (%)**			**0.042**
**College**	211 (68.3)	98 (31.7)	
**University**	770 (72.6)	290 (27.4)	
**Master degree**	305 (70.3)	129 (29.7)	
**Doctor degree**	53 (59.6)	36 (40.4)	
**Career, n (%)**			**0.001**
**Doctors**	404 (66.8)	201 (33.2)	
**Technicians**	110 (67.9)	52 (32.1)	
**Nurses**	709 (75.2)	234 (24.8)	
**Others**	78 (62.4)	47 (37.6)	
**Administrator**	43 (74.1)	15 (25.9)	
**BMI, Kg/m**^**2**^, **means (SD)**	22.04 (3.09)	22.46 (3.29)	**0.013**
**BMI group, n (%)**:			0.095
**Normal weight**	1073 (71.8)	422 (28.2)	
**Over weight**	234 (69.6)	102 (30.4)	
**Obesity**	52 (61.2)	33 (38.8)	
**Experience of wearing and taking off protective clothing in isolation ward, n (%)**:			**< 0.001**
Yes	1119 (73.5)	403 (26.5)	
No	240 (60.9)	154 (39.1)	
**Voluntarily working in the isolation ward, n (%)**:			**—**
Yes	1359 (70.9)	557 (29.1)	
No	0	0	
**History of participation training previous, n (%)**:			**—**
Yes	1359 (70.9)	557 (29.1)	
No	0	0	
**Working times, median (min, max)**:	7 (0, 37)	6 (0, 38)	0.210

### Multivariate analysis of factors associated with unqualified training outcomes

After adjusting for age group, sex, occupation, and other covariates that were statistically significant in the univariate analysis, we found that sex, educational level, occupation, and previous experience were the independent factors influencing training qualification outcomes in the multivariate analysis. The unqualified rate in female participants decreased by 27% compared to male participants; OR 0.73 (95% CI: 0.56–0.96, *P* = 0.023). Individuals with a bachelor’s degree (OR = 0.68, 95% CI: 0.51–0.92, *P* = 0.012) and master’s degree (OR = 0.61, 95% CI: 0.40–0.92, *P* = 0.019) had lower odds of being unqualified than those with a college education. Doctorate holders did not show any significant differences. When analyzed by profession, nurses were 35% less likely to be unqualified than physicians (OR = 0.65, 95% CI: 0.46–0.91, *P* = 0.013). Technicians and administrators did not demonstrate any significant differences. In addition, those lacking prior experience were 1.77 times more likely to fail than those with experience; OR 1.77 (95% CI: 1.37–2.28, *P* < 0.001). The relationship between BMI and unqualified rate disappeared after adjustment (Table 4).

**Table 4:** Associated factors of unqualified training in multivariate analysis

Factors	References	OR (95%CI)	*P*
**Sex**:	Men		
**Women**		0.73 (0.56, 0.96)	**0.023**
**Age group**:	< 30 yrs		
**31–40 yrs**		1.00 (0.77, 1.32)	0.983
**41–50 yrs**		0.94 (0.66, 1.35)	0.748
**51–60 yrs**		0.69 (0.39, 1.21)	0.195
**Education levels**	College		
**Bachelor degree**		0.68 (0.51, 0.92)	**0.012**
**Master degree**		0.61 (0.40, 0.92)	**0.019**
**Doctor degree and over**		0.76 (0.42, 1.36)	0.352
**Occupation**	Nurse		
**Doctor**		1.60 (1.12, 2.28)	**0.010**
**Technician**		1.33 (0.86, 2.07)	0.197
**Others**		1.83 (1.14, 2.94)	**0.012**
**Administrator**		0.82 (0.39, 1.74)	0.610
**Previous experiences**	Yes		
**No**		1.77 (1.37, 2.28)	**< 0.001**
**BMI**	—	1.02 (0.98, 1.06)	0.362

## Discussion

The present study contributes new insights into the determinants of successful clinical skills training for infectious diseases among medical staff in China. One of the novel findings is the significant gender disparity in qualification rates, with women showing higher qualification rates than men. This observation suggests the need for further investigation into gender-specific learning styles and potential biases in training methods. Additionally, this research emphasizes the crucial role of educational level in training effectiveness, revealing that higher education levels correlate with better qualification outcomes up to a certain point. This plateau effect at the doctoral level is a novel finding, indicating a threshold beyond which additional education does not significantly enhance training outcomes. Furthermore, the pronounced impact of professional background and previous experience on training success has not been extensively documented in the context of infectious disease clinical skills training in China. This study provides empirical evidence that these factors significantly influence training outcomes, suggesting that training programs should be tailored to leverage the specific strengths and address the needs of different professional groups.

In this investigation into the determinants of clinical skills training outcomes for infectious diseases, one of the most notable findings is the observed gender disparity in qualification rates, with female participants demonstrating higher success rates than their male counterparts. This result is particularly important given the absence of direct precedents in the existing literature, specifically regarding the impact of gender on qualification rates in clinical skills training within the context of infectious diseases.

As of October 2022, the ratio of male to female staff at this hospital is approximately 1:1.5. In this study, the female participants accounted for 65.7%, which is similar to the ratio of men to women among this hospital’s medical staff during the same period. Thus, the significance of this finding lies not only in its contribution to the broader discourse on sex differences in medical education but also in its potential implications for the design and delivery of training programs on clinical skills. It is essential to delve deeper into the causes of observed sex differences as a result of inherent learning style preferences, nature of training contents, assessment criteria, or other sociocultural factors. These insights will help develop more targeted training approaches to suit the different needs of all learners.

Previous studies suggested that educational level significantly influenced the effectiveness of clinical skill training for infectious diseases [25, 26]. The current findings revealed that participants with higher academic qualifications, particularly those with master’s degrees, were less likely to achieve unqualified outcomes, suggesting that advanced education enhances training efficacy [27, 28]. Intriguingly, this trend did not extend to doctorate holders, for whom no significant advantage was observed. This suggests a plateau effect in the correlation between educational level and training effectiveness, where beyond a certain threshold, additional academic achievement does not necessarily translate into improved training outcomes [29]. The present findings regarding the influence of educational level on qualification rates in clinical skills training for infectious diseases offer significant insights into the role of academic background in medical training effectiveness.

The current findings also demonstrate that nurses and physicians exhibited the highest qualification rates, suggesting that the nature of their professional training and routine clinical exposure significantly contributes to their success in scenario-based simulation teaching. This is consistent with the literature indicating the importance of practical experience in enhancing learning outcomes in clinical education [30, 31]. While other medical technicians were included in the current study (such as lab technicians, radiology technicians, and pharmacists), this study did not include support workers, such as nursing assistants, cleaners, and logistics support staff, whose supportive roles are also critical in keeping hospitals running, especially during infectious disease outbreaks. The training outcomes for these staff members are equally important, as they ensure a holistic approach to infection control and patient safety. Our future research will provide insights into the effectiveness of tailored training modules designed to meet the diverse needs of the entire hospital staff, including auxiliary personnel, thereby ensuring that training programs are inclusive and comprehensive.

The significant role of professional background in training effectiveness can be attributed to the differential focus of professional training pathways. For instance, nursing education typically emphasizes practical skills and patient care, potentially providing a foundation that aligns closely with the competencies assessed in scenario-based simulations. Similarly, the rigorous clinical training undertaken by physicians, focusing on diagnosis and treatment within complex clinical scenarios, may equip them with the adaptability and critical thinking skills necessary for success in simulation-based training environments. Moreover, these results highlight the pivotal role of prior experience, with individuals possessing previous exposure to protective clothing and infection control practices achieving superior qualification rates. This finding underlines the value of experiential learning and its contribution to the development of clinical skills, echoing previous research that has demonstrated the benefits of prior hands-on experience in simulation-based training outcomes [31]. It further suggests that familiarity with the practical aspects of infection control and protective equipment use, gained through real-world experience, can enhance performance in training scenarios designed to mimic clinical realities closely.

The current findings have potential implications for the development and optimization of infectious disease clinical training protocols in China. Firstly, the notable gender disparity in qualification rates underscores the importance of considering gender factors more meticulously in training protocols to ensure that training content and methodologies equally meet the needs of healthcare professionals of different genders. The significant impact of educational level on training effectiveness suggests that training protocols should include multi-level content that caters to both beginners and individuals with higher educational achievements, thereby enhancing the overall effectiveness of the training. Furthermore, the influence of professional background and prior experience on training outcomes highlights the necessity for personalized training pathways. Specifically, designing dedicated training modules for healthcare professionals from different backgrounds—such as providing more practical operation training for nurses and more clinical decision-making training for physicians—could more effectively improve their competencies in facing infectious disease challenges. Lastly, this study’s support for the effectiveness of scenario-based simulation teaching in clinical skills training for infectious diseases suggests that health management departments and medical education institutions should adopt this method more broadly in future training plans. In particular, in response to public health emergencies like COVID-19, this could rapidly and effectively enhance the clinical handling capabilities and adaptability of healthcare professionals. Therefore, the results of this study not only provide empirical support for improving existing clinical training protocols for infectious diseases but also guide the formulation of more comprehensive, personalized, and efficient training strategies. This is crucial for enhancing the capability of China and the global community in responding to infectious diseases.

### Limitations

This study has several limitations. First, selection bias may have existed. Because the study was conducted on a voluntary basis rather than using random stratified sampling, although the response rate was high, it may have attracted participation from specific medical personnel with a particular interest or experience in infectious disease management, which may not be representative of all health care workers. This could affect the generality of our findings in other populations. Second, the evaluation criteria of this study are formulated according to the two standards promulgated by the state. The analysis of the reliability and validity of the evaluation tools were not performed in the special period of the outbreak of the new champions league, because the time is pressing. This may affect the robustness and accuracy of the results. In addition, other potential biases such as information bias and measurement bias may affect our findings, which may result from the self-reported nature of some data and potential variability in how assessments are conducted by different evaluators. Such biases can be mitigated in future studies by using random sampling techniques and improving the objectivity of training evaluations. Additionally, the study’s focus on a single hospital setting may limit the applicability of the findings across different healthcare contexts with varying resources and training protocols. Accordingly, multi-center studies are needed. Finally, cross-sectional studies can only analyze factors that may be related but cannot conduct a causal association analysis.

## Conclusion

This study unveiled critical insights into factors affecting the qualification rates of medical staff undergoing COVID-19 clinical skills training based on scenario simulation in China, with a particular focus on gender, educational level, professional background, and prior experience. Notably, it found that female participants, individuals with higher educational levels up to a certain threshold, and those with specific professional backgrounds and prior experience demonstrated higher qualification rates. These findings emphasize the necessity of tailoring clinical skills training programs to the diverse needs of healthcare professionals. By adapting training methodologies to account for these variables, medical educators can enhance the effectiveness of training, thereby better preparing healthcare professionals to manage infectious diseases and improving patient care outcomes. This study underlines the importance of an inclusive, evidence-based approach to clinical skills training in the dynamic field of infectious disease management.

## Data Availability

The datasets used and/or analysed during the current study are available from the corresponding author on reasonable request.
